# Protein Charge Neutralization Is the Proximate Driver Dynamically Tuning Reflectin Assembly

**DOI:** 10.3390/ijms25168954

**Published:** 2024-08-17

**Authors:** Robert Levenson, Brandon Malady, Tyler Lee, Yahya Al Sabeh, Michael J. Gordon, Daniel E. Morse

**Affiliations:** 1 Life Sciences, Soka University of America, Aliso Viejo, CA 92656, USA; 2 Department of Molecular, Cellular and Developmental Biology, University of California, Santa Barbara, CA 93106-5100, USA; 3 Department of Chemical Engineering, University of California, Santa Barbara, CA 93106-5080, USA

**Keywords:** reflectins, intrinsically disordered proteins, protein assembly, biomaterials

## Abstract

Reflectin is a cationic, block copolymeric protein that mediates the dynamic fine-tuning of color and brightness of light reflected from nanostructured Bragg reflectors in iridocyte skin cells of squids. In vivo, the neuronally activated phosphorylation of reflectin triggers its assembly, driving osmotic dehydration of the membrane-bounded Bragg lamellae containing the protein to simultaneously shrink the lamellar thickness and spacing while increasing their refractive index contrast, thus tuning the wavelength and increasing the brightness of reflectance. In vitro, we show that the reduction in repulsive net charge of the purified, recombinant reflectin—either (for the first time) by generalized anionic screening with salt or by pH titration—drives a finely tuned, precisely calibrated increase in the size of the resulting multimeric assemblies. The calculated effects of phosphorylation in vivo are consistent with these effects observed in vitro. The precise proportionality between the assembly size and charge neutralization is enabled by the demonstrated rapid dynamic arrest of multimer growth by a continual, equilibrium tuning of the balance between the protein’s Coulombic repulsion and short-range interactive forces. The resulting stability of reflectin assemblies with time ensures a reciprocally precise control of the particle number concentration, encoding a precise calibration between the extent of neuronal signaling, osmotic pressure, and the resulting optical changes. The charge regulation of reflectin assembly precisely fine-tunes a colligative property-based nanostructured biological machine. A physical mechanism is proposed.

## 1. Introduction

The color and brightness of light reflected from skin cells of the shallow-water Loliginid squids are dynamically tunable for adaptive camouflage and communication [1]. Prior research showed that acetylcholine (ACh), released from neurons emanating from the brain to enervate patches of skin cells, activates muscarinic ACh receptors, triggering a signal-transduction cascade that culminates in phosphorylation of the reflectins, which are the major constituent of the intracellular, membrane-enclosed lamellae of nanoscale Bragg reflectors in the iridocytes [2,3,4]. The resulting assembly of the reflectins triggers the efflux of water from their membrane compartments, simultaneously shrinking the thickness and spacing of the Bragg lamellae while increasing their refractive index contrast, increasing the brightness of reflectance while tuning its color across the visible spectrum from red to blue [4,5]. These effects proved to be fully reversible and repeatedly cyclable. These processes also were found to drive a corresponding dehydration of the multiple reflectin-containing vesicles within the tunable leucophores (found thus far only in the females of one species) with the heterogeneously sized pycnotic vesicles acting as bright, white, broad-band Mie reflectors [6]. Reflectin thus is seen to act as a signal-responsive molecular machine that regulates an osmotic motor to tune nanophotonic reflectors.

Multiple mechanisms have been implicated [7,8,9,10,11,12], but uncertainty remains about the proximate trigger that first drives assembly. Xie and colleagues showed that small aromatic molecules including imidazole can drive assembly [7] and suggested a similar process might initiate assembly in the physiological control of photonic behavior [7]. In contrast, Izumi et al. and DeMartini et al. observed that neuronal triggering of the reflectin-mediated photonic changes requires ACh-induced site-specific phosphorylation of the reflectins, and they proposed that this phosphorylation overcomes the Coulombic repulsion of reflectin’s observed excess positive charges to permit condensation and assembly [2,4]. Levenson et al. then showed that a progressive reduction in reflectin’s positive charge—either by pH titration or by genetic engineering—drives proportional assembly of the initially disordered protein [8,11], and similar proportionality subsequently was demonstrated between the size of assembly and the extent of charge neutralization by electroreduction [13]. However, because titration and electroreduction reduced the charge of the imidazolium ions of histidine residues, and genetic engineering secondarily affected the net balance of aromatic residues, some questions remain. Accordingly, we chose to investigate the effects of charge neutralization of reflectin in greater depth.

## 2. Results

### 2.1. Titration Is a Surrogate for Phosphorylation, Driving Proportional Assembly

**Figure 1:**
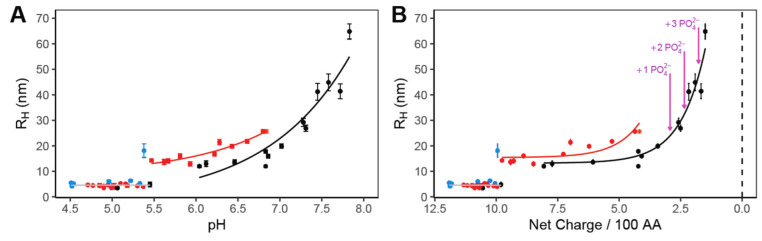
(**A**) Assembly sizes, measured by DLS, of *D. opalescens* reflectin A1 upon dilution into 25 mM buffers (blue = sodium acetate; red = MES; black = MOPS). A1 was dialyzed into 25 mM sodium acetate, pH 4.5, before pH neutralization to labeled pH conditions. Each data point corresponds to the mean of 10+ min of continuous DLS measurements of a freshly pH-neutralized sample. Error bars show the standard deviation of measurements. (**B**) Data from panel A, plotted as a function of calculated protein linear net charge density using the Henderson–Hasselbalch equation and reflectin sequence, as calculated previously [8,11]. Purple arrows indicate the calculated net charge density of reflectin A1 at pH 7 under varying degrees of phosphorylation.

As shown here by dynamic light scattering (DLS) (Figure 1A), consistent with previous demonstrations [8,11], the pH titration of reflectin’s excess positive charges serves as an in vitro surrogate for the neutralization by phosphorylation in vivo, driving the progressive assembly of the purified protein with increasing pH. The resulting assemblies are essentially stable with time. These results, from three buffer systems, demonstrate minor buffer-specific effects, likely resulting from reflectin’s sensitivity to the precise ionic strength of these buffer solutions at particular pH conditions. Using the known pK_a_ values of the constituent amino acid residues allows us to calculate the net charge density (net positive charge/100 residues) of the protein for each measured value of pH [8,11], permitting comparison of the observed effects of neutralization by titration with those that might be expected as a result of the in vivo phosphorylation with 1, 2, or 3 phosphates per reflectin (as quantitatively measured by 2D electrophoresis and mapped by LC-MS [2,14]) (Figure 1B). These results reveal a quantitatively predictive relationship between net charge density and reflectin assembly size over the range predicted for the physiological phosphorylation. They also confirm the quantitative relationship between charge and size revealed in our analyses of phosphomimetic, deletion, and other mutants [8,11].

### 2.2. Effective Neutralization by Anionic Screening Drives Assembly

**Figure 2:**
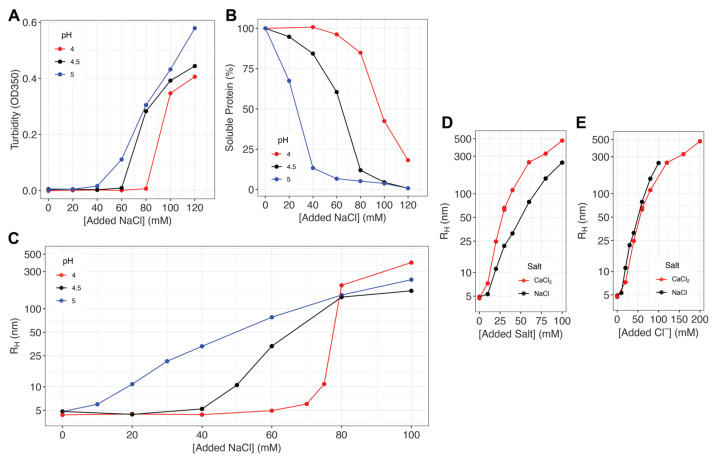
(**A**) Turbidity (OD_350_) of reflectin A1 solutions in 25 mM sodium acetate as a function of pH and NaCl concentration. (**B**) The percentage of soluble protein remaining in the supernatant after the centrifugation of samples in A is calculated relative to the amount pre-centrifugation. (**C**) Sizes of majority populations of assembled reflectin assemblies as measured by DLS. Each data point is an average of 40 min continuous DLS measurements (see Methods). (**D**,**E**) Comparison of NaCl^−^ and CaCl_2_-induced assembly at pH 5.0 as a function of (**D**) salt concentration and (**E**) Cl-anion concentration. All experiments used 10 µM reflectin A1 filtered in 25 mM sodium acetate buffer pre-titrated to designated pH. The results shown are typical results from duplicate experiments.

Although the pH dependence of reflectin assembly has been extensively characterized, the effects of ionic strength have not been. We show here that the assembly of reflectin also is driven by anionic screening of its positive charges, resulting in an effective neutralization of electrostatic repulsion that is similar to, and interacts with, the effect of pH titration (Figure 2). For these experiments, purified reflectin A1 was extensively equilibrated by dialysis into 25 mM sodium acetate buffers at pH 4.0, 4.5, and 5.0; the results of the DLS analyses below show that reflectin remains stably monomeric under these acidic conditions. Dilution into the corresponding buffers containing varying concentrations of NaCl then triggered the appearance of turbidity (measured as absorbance at 350 nm) at pH-dependent salt concentration thresholds with these assembly-inducing salt concentration thresholds decreasing as the pH is increased (Figure 2A).

Centrifugation analyses confirmed that the turbidity was due to the formation of precipitable protein assemblies (Figure 2B). Virtually all reflectin was removed by centrifugation of the precipitate at higher salt concentrations and pH, demonstrating assembly of the bulk population in accord with the turbidimetric data. Measurement of these samples by DLS confirmed assembly above the threshold salt concentrations, showing the size of these reflectin assemblies increasing reproducibly and progressively with salt concentration (Figure 2C). These salt-driven assemblies of tunable size consisted of single majority populations as judged by DLS with minority populations of larger particle sizes occasionally observed at lower salt concentrations. The majority populations were stable over ≥40 min suggesting that they undergo dynamic arrest similar to that undergone by assemblies formed through pH neutralization (cf. below). Below the salt concentration thresholds for assembly, DLS shows a majority population of stable particles of R_H_ = 4–5 nm, indicating the stability of the unstructured monomers under these conditions.

Significantly, twice as much monovalent NaCl is required to drive the same extent of reflectin assembly as driven by divalent CaCl_2_ (Figure 2D,E), showing unequivocally that it is the increasing concentration of the anion (Cl^−^) that is driving assembly by progressively screening (effectively neutralizing) the effect of reflectin’s excess positive charges.

**Figure 3:**
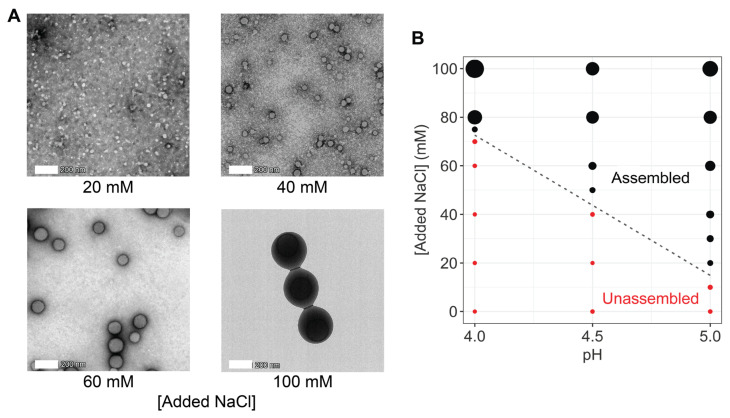
(**A**) Transmission electron microscopy (TEM) images of negatively stained A1 in 25 mM acetate, pH 5.0, assembled in the presence of labeled NaCl concentrations. The 20 mM and 40 mM NaCl panels used 1 µM A1, while 60 mM 100 mM panels used 10 µM A1. (**B**) Reflectin A1 assembly as a function of pH and NaCl concentration. Point sizes are scaled to particle size as measured by DLS. For this panel, all particles with R_H_ > 7.5 nm are considered assembled.

Transmission electron microscopy analysis of negatively stained reflectin A1 at pH 5.0 confirms the formation of assemblies with spherical morphologies exhibiting progressively larger sizes with progressively higher salt concentrations (Figure 3A).

The plotting of assembly size as a function of both pH and NaCl concentration shows that the effects of charge screening and pH are interdependent with both the salt concentration threshold and range for tunable assembly varying systematically with pH (Figure 3B). The progressively lower threshold concentration for assembly at progressively higher pH is readily explained by the progressive neutralization of the protein with increasing pH, thus reducing the requirement for neutralization by charge screening. TEM analyses of negatively stained reflectin A1 at pH 5.0 confirm the formation of assemblies with spherical morphologies exhibiting progressively larger sizes with progressively higher salt concentrations, which is consistent with assemblies formed by pH-neutralized reflectin A1 wild type and mutants [8,11].

### 2.3. Reflectin Assemblies Are Stabilized by Dynamic Arrest

**Figure 4:**
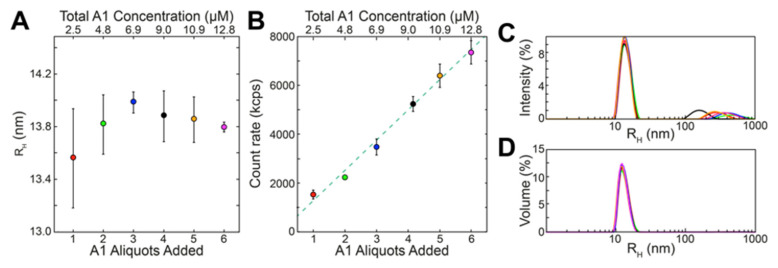
Reflectin assemblies are dynamically arrested and stable, as seen in these results of incremental additions of reflectin A1 monomers to the same 5 mM MOPS, pH 7.5 (neutralizing, assembly-driving) solution. (**A**) Reflectin assembly sizes (measured as the predominant DLS volume distributions; cf. Methods) as a function of monomer aliquots added. (**B**) Total scattering count rate as a function of monomer aliquots added (bottom x axis) and cumulative concentration (top x axis). Each point in A–B is the average of 3 replicate experiments, in each of which every aliquot addition was analyzed by 3 individual DLS measurements; error bars signify ± one S.D. between averages of replicates. Samples showed no significant variation over time following each aliquot addition, which is consistent with previous work [8,11]. Representative intensity (**C**) and volume (**D**) distributions observed after addition of the 6th aliquot of monomer with results after each aliquot shown in a different color.

The data in Figure 4 show the rapid dynamic arrest of growth and stabilization of reflectin assemblies. Incremental additions of reflectin monomer added successively to a buffered solution at pH 7.5 do not progressively augment the size of the initially formed multimers but instead assemble independently upon each new addition, forming new assemblies of approximately constant size as measured by DLS (Figure 4A). The total particle scattering signal, a function of both particle size and concentration, increases linearly with new incremental additions of protein, showing that each newly added aliquot of reflectin does not substantially aggregate with the previously added population or precipitate (Figure 4B). This also is seen by the comparison of changes in the intensity and volume distributions as a function of the incremental additions (Figure 4C,D). Significantly, the constancy of sizes indicated in the volume distribution (Figure 4D) confirms that the majority of reflectin monomers independently assemble to the same predetermined size upon each new addition to the same solution at fixed pH. Features of the reflectin sequence predisposing it to rapidly form a network of extensive, non-covalent cross-links that may contribute to such dynamic arrest are considered in Section 4.

## 3. Materials and Methods

*Reflectin expression and purification*: Recombinant *Doryteuthis opalescens* reflectin A1 was produced from a construct described previously, possessing no affinity tags [8,11]. The expression and purification of A1 were performed as described previously [8]. Briefly, Rosetta 2(DE3) *E. coli* cells (EMD Millipore, Burlington, MA, USA) were grown in LB cultures from freshly plated transformants in the presence of 50 mg/mL kanamycin. Expression was induced at A_600_ ~ 0.6 and allowed to proceed for approximately 6 h, after which cells were centrifuged and frozen at −80 °C until purification.

Reflectin A1 protein was found entirely within inclusion bodies; these were purified from thawed cell pellets with BugBuster medium (Novagen, Inc., Madison, WI, USA) as directed by the manufacturer’s instructions. Inclusion bodies were solubilized in 5% acetic acid, 8 M urea, which was followed by dialysis against 5% acetic acid, 8M urea to remove guanidinium. Proteins were purified by ion exchange over a HiTrap XL (GE Healthcare, Chicago, IL, USA) cation exchange column and eluted with a gradient of 5% acetic acid, 6M guanidinium. Fractions with reflectin were collected and diluted in 5% acetic acid, 8M urea upon which they were loaded onto a MonoS GL column (GE Healthcare, Chicago, IL, USA) and eluted with a step gradient of 5% acetic acid, 6M guanidinium concentration. Eluted reflectin was concentrated and loaded onto a reverse phase C10 column by HPLC equilibrated with 0.1% trifluoracetic acid (TFA) in H_2_O and eluted over a gradient of 95% acetonitrile, 0.1% TFA. Protein was then lyophilized and stored at −80 °C until solubilization. Purity was assessed by SDS-PAGE on 10% Tris-acetate SDS-PAGE gels (Life Sciences, Carlsbad, CA, USA).

*Protein solubilization and sample preparation*: To perform the pH neutralization assays, lyophilized A1 was solubilized by the addition of 0.22 μm filtered H_2_O. Concentration was determined by measuring A_280_ using a calculated extinction coefficient of 120,685 M^−1^ cm^−1^. For DLS assays, the protein was then diluted to 81 μM for use as a stock solution. All buffers and water were filtered upon preparation and centrifuged at 18,000× *g* for 10 min promptly before use and equilibrated to room temperature. The protein was stored at 4 °C between use.

To evaluate the effects of incremental additions of reflectin at a given pH, increments of 1.25 μL of 81 μM H_2_O-solubilized A1 WT aliquots were added to 40 μL starting neutralizing buffer, 5 mM MOPS pH 7.5, within the DLS cuvette. Samples were measured immediately and continuously after the addition of protein for ≥10 min, after which the next aliquot was added and measured. Total scattering count rates were obtained from the derived count rates as calculated by the instrument software.

For salt-driven assembly, purified reflectin A1 was first solubilized in 25 mM sodium acetate, pH 4.5 and then dialyzed 1000-fold three times into 25 mM sodium acetate pH 4.5 buffer to fully equilibrate the protein, after which the protein was either used or dialyzed 1000-fold three times into 25 mM sodium acetate buffer at pH 4.0 or 5.0. Reflectin A1 showed no signs of assembly or precipitation during this exchange process. The concentration of reflectin A1 was then determined spectrophometrically, after which the protein was diluted to make a 100 μM stock solution. This stock was then diluted 10x into a pre-equilibrated sodium acetate and salt solution of appropriate pH to a final concentration of 10 μM. BSA controls were prepared from pure lyophilized BSA (Thermo Fisher Scientific, Waltham, MA, USA) solubilized into 25 mM sodium acetate, pH 4.5, and then identically prepared as described above.

*Dynamic light scattering*: DLS was performed using a Zetasizer Nano ZS (Malvern Panalytical, Worcestershire, UK). Measurements were performed using 40 μL sample volumes at 25 °C. Samples were measured continuously, immediately after assembly, to ensure sample equilibration and stability. At least 3 DLS measurements were performed per replicate.

*Turbidity and Centrifugation*: Turbidity and centrifugation assays of pH-neutralization driven assemblies were reported previously [8]. For the salt-driven assemblies, the protein was diluted into 25 mM sodium acetate and salt solutions to yield 40 μL of 10 μM A1. After a 10-min incubation period at room temperature, A_350_ was measured directly using a Nanodrop spectrophotometer (Thermo Fisher Scientific, Waltham, MA, USA). Samples were then centrifuged for 10 min at 18,000× *g*, and the supernatant A_280_ was measured to determine the protein remaining compared to a zero-salt solution in otherwise identical buffer conditions.

*Transmission Electron Microscopy*: Salt-driven assemblies were initially prepared at 10 μM A1 concentrations and then either directly applied (for 60 mM and 100 mM NaCl) to 400-mesh carbon-coated grids (Electron Microscopy Services, Hatfield, PA, USA) or diluted 10X into buffer and then applied (20 and 40 mM NaCl). Prior to sample application, grids were treated with glow discharge for 20 s. Then, 5 μL of freshly prepared sample was applied for 2 min before wicking away the excess with filter paper. Samples were then negatively stained by the three-time application of 20 μL freshly filtered 1.5% uranyl acetate, with wicking in between each application. Samples were visualized with a Talos G2 F200X TEM (Thermo Fisher Scientific, Waltham, MA, USA).

## 4. Discussion

Previous X-ray scattering analyses confirm the sphericity, size and low polydispersity of reflectin’s assemblies [15]. The data presented here show that charge screening by added salt anions—neutralizing the effect of charge while leaving the imidazolium ions intact—drives the assembly of reflectin in a manner closely comparable to the previously reported effects of pH titration [8,11], electroreduction [13,16], and genetic engineering [8,11], and that the effects of salt concentration and pH are interdependent. Analyses of assembly driven by pH titration (Figure 1) show comparable results with three buffer systems, ruling out any major effects of the buffers used. (The small discontinuity at ca. pH 5.5, which is observed consistently [11], is formally indicative of an energy barrier to initial assembly.) Small buffer-dependent differences in assembly sizes are observed, which are consistent with reflectin’s sensitivity to ionic strength. Analyses in terms of reflectin’s effective repulsive net charge densities confirm the previously reported, predictive and precisely proportional relationship between the extent of net charge density neutralization and the size of the resulting reflectin assemblies, and they demonstrate that these observations are consistent with those predicted for the physiologically observed phosphorylation [2,11] of reflectin.

Our results demonstrate that the charge neutralization of reflectin A1 is the proximate trigger of assembly and that the extent of charge neutralization proportionally controls the size of the resulting assembly. This precise relationship depends upon the rapid dynamic arrest and subsequent stability of assembly size. At low pH and low salt concentrations, condensation, folding, and assembly are all inhibited by electrostatic repulsion between cationic side chains, which is present in high proportion in the reflectins [8,9,17]. A reduction in this repulsion by progressive charge-neutralizing phosphorylation in vivo, or by pH titration, charge screening, electroreduction [13,16] or genetic engineering [11] in vitro, allows attractive chain interactions to quickly drive condensation, folding and assembly with rapid arrest by a network of interactions that inhibits further dynamics and growth. Recent experimental evidence [18] and computational simulations [15] demonstrate that this dynamic arrest of assembly is the direct result of the continually tunable balance between short-range (weak) attractive forces and long-range (strong) repulsive forces, as well understood for many colloidal systems [19], and that although the size of the assemblies remains constant for each set of conditions, they actually are in dynamic equilibrium with monomers from the surrounding medium. In the case of reflectin assembly, these weak attractive forces are likely to include a combination of hydrogen and hydrophobic bonding, β-stacking, coil–coil interactions, cation–π, sulfur–π, π–π, van der Waals and other forms of non-covalent bonding, while reflectin’s finely tunable Coulombic repulsion likely comprises the dominant strong interaction [9,11,15].

**Figure 5:**
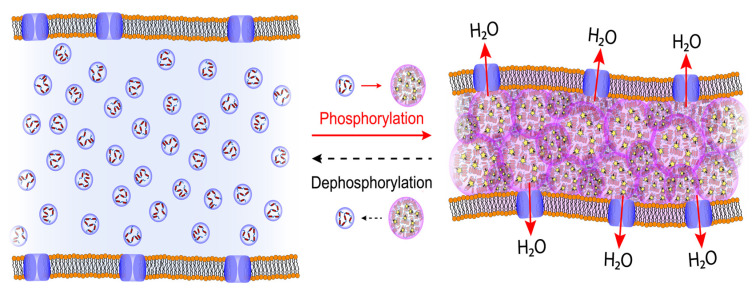
Charge neutralization by phosphorylation (stars) triggers a precisely limited assembly of the cationic, block-copolymeric protein, reflectin, driving a calibrated osmotic tuning of color reflected from Bragg lamellae in cells of squid skin. Effective neutralization by anionic screening provides a surrogate for phosphorylation, driving reflectin’s proportional assembly in vitro.

This precise relationship between effective charge and size has a profound, direct bearing on the physiological mechanism by which reflectin controls biophotonic behavior (Figure 5). Because the size of the reflectin multimers is directly and inversely proportional to the *number concentration of reflectin particles* within their membrane-bounded compartment, the charge vs. size relationship directly controls the osmotic pressure within that compartment and thus precisely controls the osmotic dehydration of that compartment, driving the observed changes in the wavelength and intensity of the reflected light. We note that our data indicate that reflectins are expected to be in an assembled state at the pH and salt concentrations within a typical physiological system. We therefore hypothesize that the native tunable physiological system does not function by simply switching assembly on and off through modulated reflectin (de)phosphorylation but rather via a subtler mechanism that tunes the mean sizes of reflectin assemblies. This hypothesis is in concordance with the calculations shown in Figure 1b, showing that the phosphorylation of reflectin assemblies would be expected to tune assembly size rather than simply inducing assembly. The hypothesis for such a dynamic physiological milieu is consistent with the dynamic colloidal systems described above [15] and (b) the growing recognition and understanding of biomolecular condensates, which are liquid-like compartments greatly concentrated in biomolecules in physiological systems [20,21]. The dependence of osmotic pressure on the extent of phosphorylation of reflectin thus ensures a precisely calibrated relationship between the neuronal delivery of ACh and the resulting color and brightness of the iridocyte. Two other mechanisms contribute to the signal-dependent dehydration of the Bragg lamellae: “dewatering” of the reflectin molecules by steric exclusion resulting from the induced folding and hierarchical assembly of the protein, and the Gibbs–Donnan re-equilibration resulting from the similar steric displacement of some of the protein’s neutralizing small counterions, the transmembrane migration of these ions to maintain electrical neutrality, and the consequent movement of water to equalize osmotic pressure [4]. Both mechanisms would operate in strict proportionality to the extent of assembly as well.

We previously discussed the detailed mechanisms controlling reflectin’s assembly after activation by charge neutralization with data indicating that neutralization of the block copolymeric protein’s cationic, Coulombic repulsion permits sequential condensation and folding with the emergence of previously cryptic hydrophobic surfaces that are likely to facilitate hierarchical assembly [2,8,9]. The block copolymeric structure of reflectin A1 can thus be thought of as a concatenation of alternating, opposing expansion and contraction springs: Coulombic repulsion of the cationic linkers (expansion) keeps the molecule in an extended and intrinsically disordered state until charge neutralization sufficiently opposes that repulsion, relaxing the stress on the conserved domains to allow the entropic drive encoded in their sequences to trigger condensation and secondary folding (contraction), causing an emergence of hydrophobic surfaces and/or beta structures that facilitate hierarchical assembly [8,9,22]. Reflectins have also been shown to bind and drive the agglomeration of synthetic vesicles with compositions similar to cellular membranes, suggesting that reflectin–membrane interactions may play an important role in the formation and/or tunability of the physiological Bragg lamellae [23].

Beyond their physiological roles in biophotonics, reflectin proteins have been shown to form materials of various structures including optical gratings, fibers, thin films, and hydrogels, all with properties responsive to their environment [24,25,26,27,28,29,30,31]. They have also been shown to serve as substrates for cell growth [32] and act as proton conductors and transistors [33,34,35].

It is interesting to note that the repeated anionic domains in an intrinsically disordered melanosomal protein recently have been found to act as the charge sensors controlling acid-triggered amyloidogenic assembly [36], which is analogous to the role of the repeated cationic linkers that act as the charge sensors controlling reflectin’s assembly [11]. While numerous examples of pH- and charge-regulated transitions of protein assembly states are known [37,38,39,40,41], and this principle recently was used to design synthetic peptides with multiple histidines buried with hydrogen-bonded networks to realize preprogrammed, pH-driven conformational changes [42], this is the first report of which we are aware of a genetically preprogrammed, precisely controlled dependence of the size of protein assembly upon net charge density with direct connection to the mechanism of control of biological function in vivo.

## 5. Conclusions

Charge neutralization by phosphorylation triggers a precisely limited assembly of the cationic, block-copolymeric protein, reflectin, to drive a calibrated osmotic tuning of color reflected from Bragg lamellae in cells of squid skin [2,3,4,5,6,8,11]. Dynamic light scattering analyses show here that effective neutralization by anionic screening provides a surrogate for phosphorylation, driving reflectin’s proportional assembly in vitro. Because anionic screening is sufficient to drive reflectin assembly with no change in the protein’s histidine imidazolium ions or other aromatic residues, we conclude that charge neutralization and its consequent neutralization of Coulombic repulsion alone are the proximate driver of assembly with no requirement for additional aromatic compounds as suggested by Xie et al. [7]. Assembly driven by effective neutralization by charge screening and by pH titration of the protein’s imidazolium ions of histidine residues are interdependent, as expected, and the calculated effects of phosphorylation in vivo are shown to be consistent with these effects observed in vitro. The precise proportionality between the extent of charge neutralization and assembly size is shown to result from the rapid dynamic arrest of assembly and subsequent stability, which is the result of a continual equilibrium tuning of the balance between the protein’s finely tunable Coulombic repulsion and its multiple short-range attractive interactions, ensuring precise calibration between the initiating neuronal signal triggering reflectin phosphorylation [2,6] and the resulting osmotically fine-tuned color reflected from the reflectin-containing Bragg reflectors in vivo. A physical mechanism for the continually fine-tuned control of reflectin’s assembly by charge, considering the protein’s alternating block-copolymeric domains acting analogously to a concatenation of alternately opposing springs, is discussed.

## Data Availability

The data are available from the corresponding authors upon reasonable request.
